# Arsenic on the Hands of Children

**DOI:** 10.1289/ehp.113-a364a

**Published:** 2005-06

**Authors:** John C. Kissel

**Affiliations:** Department of Occupational and Environmental Health Sciences, University of Washington, Seattle, Washington, E-mail: jkissel@u.washington.edu

Kwon et al. (2004) reported significantly elevated dislodgeable soluble arsenic loads on (one or both?) hands of children following play on structures treated with chromated copper arsenate (CCA) but then concluded that the observed difference is unimportant:

With a safe conservative assumption that all the arsenic on children’s hands is ingested, the measured value is below the estimated average daily intake of inorganic arsenic from water and food ….

However, Kwon et al.’s analysis is not conservative for at least two reasons. First, it is likely that they substantially underestimated arsenic on hands. Kwon et al. reported, but apparently did not actually measure, total arsenic on hands. They washed hands, filtered the wash water, and measured soluble arsenic in the filtrate. Insoluble residue was measured as dry mass gain on the filters. They then estimated insoluble arsenic on hands as the product of the average arsenic concentration in playground sand samples (not solids recovered from hands) and filter dry weight gain. I did not arrive at this conclusion because the procedures are clearly described in the paper but because *a*) there is no discussion of extraction of filters and *b*) the ratios of minimum, mean, median, and maximum “sand arsenic” on hands to minimum, mean, median, and maximum sand mass recovered from hands are nearly constant and equivalent (in all cases but one) to the mean concentration reported for each playground.

This procedure could easily give a very poor estimate of insoluble arsenic on hands because unfractionated 0- to 6-in sand samples are likely to be a poor surrogate for adherent particles. The filter residue from the hand-wash water probably contained at least some wood particles with much higher arsenic concentrations and lower densities than the playground sand. Hemond and Solo-Gabriele (2004) reviewed studies in which (typically adult) human hands were used to deliberately wipe CCA-treated lumber and reported much higher arsenic residues on hands than found by Kwon et al. (2004). One obvious potential explanation is that the arsenic concentration in material dislodged from CCA-treated wood (Nico et al. 2004) can easily be 1,000-fold higher than the 2–3 ppm found by Kwon et al. in playground sand.

Second, the observed loads that Kwon et al. (2004) reported may be greatly influenced by the very activity they wish to assess. That is, mass recoverable at any given time reflects net accumulation and does not include material already ingested. Consider the following simplified model of mass accumulation on hands:


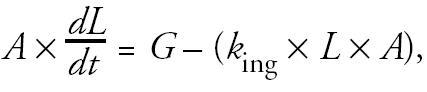


where *A* = area (in square centimeters), *L* = load (in milligrams per square centimeter), *G* = net gain in the absence of ingestion (addition minus losses other than ingestion; in milligrams per hour), and *k*_ing_ = a first order rate constant describing ingestion (per hour).

At steady state,


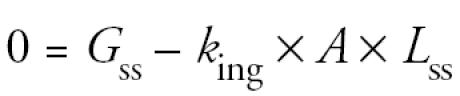


and


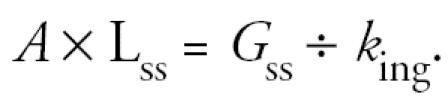


Assuming reasonable efficiency of washing, Kwon et al. (2004) provided a measure of the product of the two variables on the left hand side (for soluble arsenic). They have not measured either of the variables on the right hand side. In the absence of knowledge of *k*_ing_, they guessed. Because an infinite number of paired values of *G* and *k*_ing_ can be selected to match the available data, large values of *k*_ing_ are not excluded. Hence any reassuring conclusion based on this work is a reflection of the assumed rate at which hand residues are orally harvested and not of the reported measurements.

Kwon et al. (2004) further concluded that

Most of the arsenic on children’s hands is water soluble and is readily washed off with water. We recommend that children wash their hands after playing to reduce their potential exposure to arsenic.

Again, this conclusion is not supported by evidence presented in the article. To evaluate efficiency of washing, some measure of the initial mass present is required. Kwon et al. measured removable soluble arsenic and estimated removable insoluble arsenic. They did not measure or estimate either soluble or insoluble arsenic remaining on the hands. Because insoluble arsenic bound to soil or wood is likely to be at least partially removed mechanically by washing regardless of solubilization, washing is probably a good strategy. However, that argument is merely logical rather than empirical and could have been made in the absence of Kwon et al.’s experiments.

Kwon et al. (2004) stated that the purpose of their study was to provide “direct measurement of arsenic levels on the hands of children in contact with … CCA-treated wood ….” Given that arsenic is amenable to biomonitoring via urine, comparable urine samples from children who do and do not play on CCA-treated structures are what is most needed. Then perhaps we would be able to stop guessing about ingestion rates.
